# Factors associated with utilization of cervical cancer screening services among HIV-positive women aged 18 to 49 years at Lira regional referral hospital, Northern Uganda

**DOI:** 10.1186/s12905-024-02957-9

**Published:** 2024-02-12

**Authors:** Florence Layet, Tom Murungi, Nasser Ashaba, Eustes Kigongo, Marc Sam Opollo

**Affiliations:** 1 Faculty of Public Health, Lira University, Lira City, Uganda; 2 Department of Midwifery, Faculty of Nursing and Midwifery, Lira University, P.O Box 1035, Lira City, Uganda

**Keywords:** Cervical Cancer, Cancer Screening, HIV, Factors, Utilization

## Abstract

**Background:**

Women with HIV have a higher risk of getting cervical cancer due to induced immunosuppression. Though this burden could be avoided through early identification and appropriate management, there is a paucity of information about the utilization of cervical cancer screening (CCS) services in Lira City, Uganda. This study investigated the level and factors associated with the utilization of cervical cancer screening services among HIV-positive women aged 18 to 49 years at Lira Regional Referral Hospital, Lira City, Uganda.

**Methods:**

We conducted a facility-based cross-sectional study employing quantitative techniques. We used consecutive sampling to recruit 297 HIV-positive women at the ART clinic of Lira Regional Referral Hospital. A structured researcher-administered questionnaire was used to collect data. Descriptive statistics were performed to summarize the data. A modified Poisson regression using robust standard errors was performed to ascertain the factors associated with the utilization of cervical cancer screening. Prevalence ratios at 95% confidence intervals were reported.

**Results:**

Out of 297 respondents, 175(58.9%) utilized cervical cancer screening in this study. The factors found to be associated with CCS were; having ever heard of CCS (Adjusted Prevalence Ratio [PR] 1.80, 95% CI 1.31–2.49, *p* < 0.001), knowing where CCS is done (Adjusted PR 1.99, 95% CI 1.42–2.81, *p* < 0.001), fear of CCS outcomes (Adjusted PR 0.67, 95% CI 0.54–0.84,*p* < 0.001), not knowing whether CCS is beneficial or not (Adjusted PR 0.39, 95% CI 0.20–0.75,*p* = 0.005) and having friends/relatives who screened for cervical cancer (Adjusted PR 1.31, 95% CI 1.09–1.59, *p* = 0.005).

**Conclusion:**

The level of utilization of cervical cancer screening services among HIV-positive women was suboptimal. Implementation of structured interventions aimed at improving cervical cancer screening awareness among HIV-positive women is crucial. Additionally, to increase opportunities for screening and knowledge on cervical cancer prevention, screening programs can target HIV-positive women during their routine clinic visits.

## Background

Cervical cancer is the fourth most common cancer, and the second leading cause of cancer deaths among reproductive-aged women worldwide [1, 2]. Cervical cancer is sexually transmitted and is caused by long-term infection with certain types of human papillomavirus (HPV) [3]. The HPV types (16 and 18) cause approximately 50% of high-grade cervical pre-cancers [1]. Early first sexual intercourse, multiple sexual partners, and immune suppression cause a predisposition to HPV [4]. Cervical cancer can affect any woman; however, it is frequently common among women aged 18 to 49 years with a positive diagnosis of HIV [4]. HIV-positive women are at a higher risk of developing cervical cancer because both HIV and HPV infections are transmitted sexually, and because HIV-induced immunosuppression increases the likelihood that HPV infection will persist in these women [5, 6].

According to the World Health Organization (WHO), an estimated 342,000 women out of 604,000 diagnosed with cervical cancer died as a result of complications in 2020 [1, 7]. This disease is on the rise in Sub-Saharan Africa, with more than 75,000 new cases and 50,000 deaths each year, which is exacerbated by HIV infection [8]. The region has the highest global prevalence of HPV [9, 10]. In Uganda, nearly 6413 women were newly diagnosed with cervical cancer in 2018, with 2400 succumbing to the disease [11]. Cervical cancer incidence in Uganda is three times that of the global average and is the leading cause of death among women [11]. In 2020, the Global Cancer Observatory showed that about 35.7% of cancer cases among women in Uganda were due to cervical cancer [12]. In the same year, the prevalence of HIV among women of reproductive age was 7.1%, higher than their male counterparts, 3.8% [13]. Current projections indicate that Uganda will have about 6400 new cervical cancer cases and 4300 deaths per year by 2025 [13]. Unfortunately, the cervical cancer incidence among women living with HIV in Africa is high ranging from 13–47% [14].

In the efforts to eliminate cervical cancer, the WHO established a strategy targeting early screening and prompt management [15]. The sexually transmitted disease guidelines in Uganda also recommend annual cervical cancer screening for women living with HIV [16]. Uganda commenced cervical cancer screening in 2007 with support from WHO, PATH, and UWHI among other stakeholders, and currently, the screening and treatment strategy is being implemented [17]. Some of the screening modalities include Pap smear, HPV testing, and visual inspection with acetic acid (VIA) [18]. The screening and treatment for cervical cancer at the health facilities is done by nurses and midwives. However, studies have reported that the screening program Uganda implements is erratic, opportunistic, and present in some places due to a lack of financial resources and commitment [19]. This is likely to compromise the achievement of the target of screening 70% of women by high-performance tests by 35 years and again by 45 years of age [20]. However, there is poor integration of cervical cancer screening services into routine HIV care [21].

There is limited research on the utilization of cervical cancer screening among women living with HIV with the prevalence ranging from 33% in urban healthcare centres [22] to 43.75% in Gulu district [23]. Many predictors have been reported to influence the low uptake of screening including poor accessibility, poor awareness, and limited resources for conducting screening by the health facilities [19, 21, 23]. There is limited research on cervical cancer screening among women living with HIV in Lira district. At Lira Regional Referral Hospital (LRRH), HIV treatment services have not been integrated with cervical cancer screening services though HIV care services have been incorporated within other programs such as maternal and child health. This could also lead to missed opportunities for screening despite provider recommendations and the availability of free screening services. Thus, we investigated the factors associated with the utilization of cervical cancer screening services among HIV-positive women at Lira Regional Referral Hospital (LRRH), Lira City.

## Methods

### Study design

This was a cross-sectional facility-based study that used quantitative methods of data collection conducted in April 2023. Data was collected from Lira Regional Referral Hospital (LRRH) situated in Lira City, Northern Uganda.

### Study site

The study was conducted at Lira Regional Referral Hospital, the referral hospital for the districts of Amolatar, Apac, Kwania, Dokolo, Lira, Kole, Otuke, Alebtong and Oyam. It is located approximately 339 km (211 mi), by road, north of Kampala Capital City. The hospital offers general services as well as specialist clinical services. With about 12,000 people attending LRRH, about 800 women aged 18–49 years are enrolled in Antiretroviral Therapy (ART), receiving care from the ART clinic, units with integrated HIV services, and community drug distribution points. Lira Regional Referral Hospital was specifically selected for this study because the level of utilization of cervical cancer screening services among HIV-positive women is unknown despite the presence of those services at the facility.

### Study population

The target population was HIV-positive women aged 18 to 49 years in Lira City and the accessible population was the HIV-positive women aged 18 to 49 years receiving care at LRRH. This age group was selected because cervical cancer can affect any woman, but it is frequently common among women aged 18 to 49 years with a positive diagnosis of HIV.

### Sample size and sampling

We used the Yamane (1967) formula to obtain the minimum sample size for the study. Records from LRRH indicated that 800 women aged 18 to 49 years were enrolled in ART. An error margin of 5% was used. To cater to the non-response rate, the sample size was increased by 10% to have a final sample of 297 participants. Consecutive sampling was employed to obtain the study participants. This was employed because women walk into the facility on different appointment days and times. Likewise, not all the 800 enrolled women regularly visited the clinic. Therefore, recruitment followed availability and those who met the inclusion criteria were recruited until the required sample size was achieved. Participants were recruited from the ART clinic.

### Inclusion and exclusion criteria

The study included all HIV-positive women aged 18 to 49 years registered with the ART clinic of LRRH. Those who were very ill, those who had a history of hysterectomy, and those with a known positive cervical cancer diagnosis were excluded from the study.

### Variables

The dependent variable for the study was the utilization of cervical cancer screening. This was a self-reported measure based on whether a respondent had done cervical cancer screening in the past 12 months with a Yes or No response, and measured as a proportion. The independent variables included sociodemographic factors, individual factors, and health system factors relating to cervical cancer screening.

### Data collection technique, tools and procedure

An interviewer-administered questionnaire containing close-ended questions was used to collect data. The questionnaire was developed by all the researchers after requesting and adapting questions from studies in a similar context in order not to miss out on any information [24, 25]. The questionnaire consisted of 5 sections: socio-demographic characteristics (age, religion, level of education, marital status), utilization of CCS, awareness of CCS (ever heard of CCS, know the place for screening, source of information on CCS), individual factors, and health system factors affecting utilization of CCS. Individual factors included; having friends/peers who have ever screened, ever being vaccinated for HPV, thinking screening is of any benefit, fear of screening outcomes, being embarrassed by the screening procedure, distance to the health facility, partner encouragement to screen, and partner support in receiving HIV treatment. Health system factors included; the provision of health education on screening, health facility appointments for screening, conducting outreach screening, health worker recommendation for screening, availability of screening services, and time convenience for provision of CCS services at the facility. The questionnaire was pretested among 27 women enrolled on ART at Dokolo Health Center IV in Dokolo district and a reliability coefficient of 0.78 was obtained. The questionnaire was administered by two research assistants who both had bachelor’s degrees in Midwifery. The research assistants were trained by the research team before data collection on the data collection process and ethical conduct for 3 days. Before the interviews, participants were provided with a description, of the purpose and procedures of the study to obtain their consent. Participants who consented to participate signed the consent forms or provided their thumbprints. Unique codes were assigned to the participants and assured that their information was not going to be discussed with any third parties to ensure confidentiality. Completeness check was always done by ensuring that all the questions in the tool were answered while in the field.

### Data management and analysis

Data entry was done in Microsoft Excel (2013), cleaned to remove any inconsistencies, and later checked for completeness. Data were exported to Statistical Product and Service Solutions (SPSS) version 29 software for post-entry coding and final analysis. For descriptive analysis, data was summarized as means with standard deviations, simple frequencies, and proportions. In bivariate and multivariate analysis, we utilized prevalence ratios by way of a modified Poisson regression method through the generalized linear model with Poisson family and log link without an offset, while integrating robust standard errors. This was done due to the high prevalence of the main outcome (58.9%), which could easily overestimate the effect size if the normal logistic regression were conducted. In bivariate analysis, *p* < 0.2 was considered for associations. Variables found to have associations (*p* < 0.2), and other plausible from literature were further assessed at multivariate analysis after careful examination of underlying assumptions. The backward elimination method was used to build the final model with only variables with statistically significant associations (*p* < 0.05). Prevalence ratios, corresponding 95% confidence intervals, and p-values were reported. To ensure that the generated model fitted the data, a Pearson goodness-of-fit test was performed and showed a *p* > 0.05 hence the fitness of the model.

### Ethical statement

#### Ethical approval

was sought from Lira University Research Ethics Committee (LUREC-2022-5). All the principles of engaging humans as subjects as outlined in the Declaration of Helsinki were adhered to throughout the entire period of study.

## Results

**Fig. 1:**
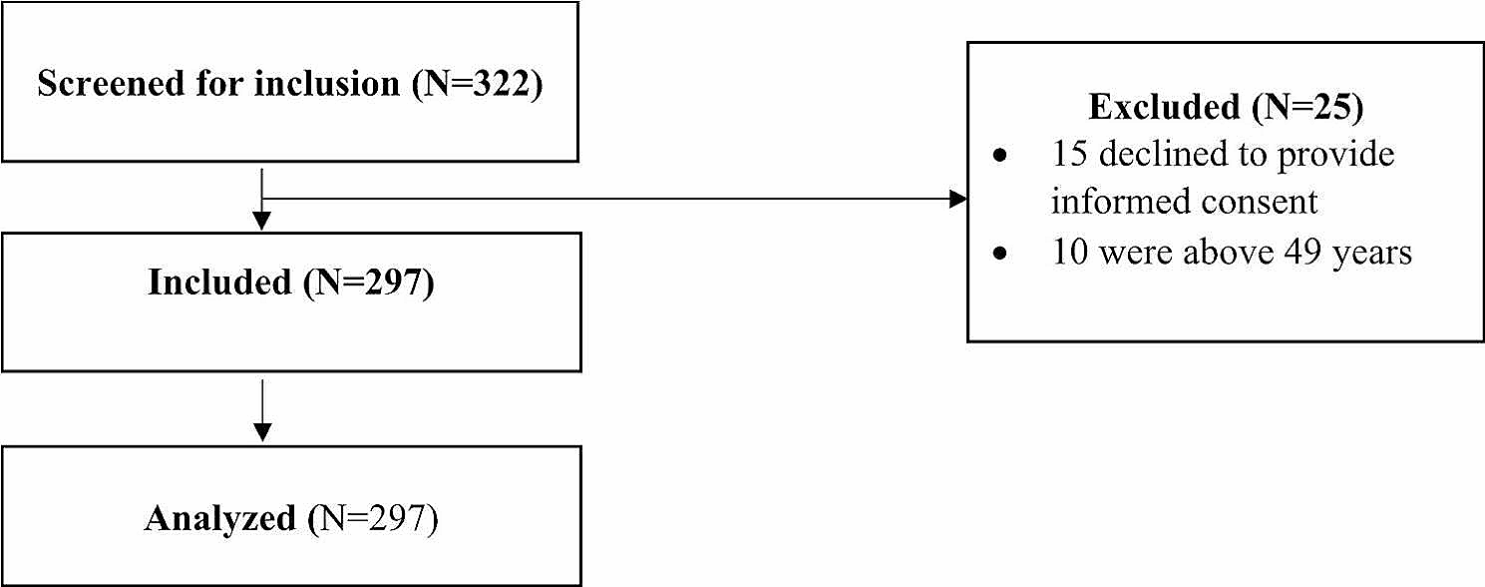
Recruitment profile of participants

Figure 1 indicates that out of the 800 eligible women enrolled in ART at Lira Regional Referral Hospital, 322 were screened for inclusion into the study, and 297 provided full information that was analyzed.

### Sociodemographic and clinical characteristics

Table 1 shows that the majority 157(52.9%) were aged 31–49 years. About half, 153(51.5%) of the participants were para 1–3 and the majority 141(47.5%) were married. The majority, 142(47.8%) of the participants had finished secondary level of education, and half, 150(50.5%) were self-employed. Most, 176(59.3%) of the participants were diagnosed with HIV/AIDS five years ago and above and the majority, 172(57.9%) had never been diagnosed with any Sexually Transmitted Infections, STIs) in the past 12 months. More than three-quarters, 242(81.5%) of the participants had only one sexual partner. Most, 256(86.2%) of the participants received information regarding cervical cancer screening from the hospital.

**Table 1:** Sociodemographic and clinical characteristics of HIV-positive women aged 18–49 years at Lira Regional Referral Hospital

Variable	Frequency (n)	Percentage (%)
**Age**		
18–30	140	47.1
31–49	157	52.9
**Parity**		
Nulliparous	44	14.8
Para 1–3	153	51.5
Para 4 and above	100	33.7
**Marital status**		
Single	20	6.7
Married	141	47.5
Cohabiting	106	35.7
Divorced	30	10.1
**Level of education**		
No formal education	18	6.1
Primary level	104	35.0
Secondary level	142	47.8
Tertiary level	33	11.1
**Occupation**		
No formal employment	101	34.0
Civil/Private servant	46	15.5
Self-employed	150	50.5
**Time of HIV diagnosis**		
Less than 5 years ago	121	40.7
5years ago and above	176	59.3
**Diagnosed with STI in the past 12 months**		
No	172	57.9
Yes	125	42.1
**Number of sexual partners**		
No partner	9	3.0
Only one partner	242	81.5
More than one partner	46	15.5
**Source of information**		
Hospital	256	86.2
Friends	145	48.8
Family members	40	13.5
Church/mosque	12	4.0
Media	63	21.2

Sociodemographic and clinical characteristics of the participants

STI-sexually transmitted infection

### Utilization of cervical cancer screening services

Out of 297 participants, more than half, 175(58.9%) had ever screened for cervical cancer in the past 12 months.

### Reasons for non-utilization of cervical cancer screening services

Among those who had not screened, the majority, 42(34.4%) reported that they didn’t have time to go for CCS, 41(33.6%) reported time inconvenience for the provision of CCS services at the facility and 39(32.0%) didn’t screen for CCS because the procedure is painful. Furthermore, 35(28.7%) reported that the CCS procedure is time-consuming and 29(23.8%) felt that they were healthy and were not willing to go for CCS.

### Factors associated with the utilization of cervical cancer screening services

Table 2 show indicates that the individual factors that were significantly associated with utilization of CCS at *p* > 0.2 were; Having ever heard of CCS, knowing where CCS is done, fear of CCS outcomes, thinking CCS is beneficial, not knowing whether screening is beneficial, and having friends/relatives who screened for cervical cancer. Health system factors that were significantly associated with CCS utilization were; the provision of health education on CCS, scheduling of appointments for CCS at the facility, conducting of CCS outreaches, provider recommendation for CCS, convenient hours for CCS, and availability of CCS services. Table 2 also shows that having ever heard of CCS (Adjusted Prevalence Ratio [PR] 1.80, 95% CI 1.31–2.49, *p* < 0.001), knowing where CCS is done (Adjusted PR 1.99, 95% CI 1.42–2.81, *p* < 0.001), fear of CCS outcomes (Adjusted PR 0.67, 95% CI 0.54–0.84,*p* < 0.001), not knowing whether CCS is beneficial or not (Adjusted PR 0.39, 95% CI 0.20–0.75,*p* = 0.005) and having friends/relatives who screened for cervical cancer (Adjusted PR 1.31, 95% CI 1.09–1.59, *p* = 0.005) were factors associated with utilization of cervical cancer screening. Women who had ever heard about CCS were more likely to have screened compared to those who had never heard about CCS. Additionally, women who knew where CCS is done were more likely to have screened compared to their counterparts who did not know where CCS is done. Women who feared the outcomes of CCS were less likely to have screened compared to those who did not fear the outcomes of cervical cancer screening. Similarly, women who were not sure whether CCS was beneficial were less likely to have screened compared to those who said it was not beneficial. Lastly, women who had friends and or relatives who had ever screened were more likely to have screened compared to those who did not.

**Table 2:** Bivariate and multivariate analysis of the factors associated with the utilization of cervical cancer screening services among HIV-positive women aged 18-49years at Lira Regional Referral Hospital, Lira City, Northern Uganda

Factors	Ever screened		Bivariate analysis		Multivariate analysis	
	No. n (%)	Yes. n (%)	**CPR (95% CI)**	**P-value**	**APR (95% CI)**	**P value**
**Ever heard of CCS**						
No	45(36.9)	24 (13.7)	1.00			
Yes	77(63.1)	151(86.3)	1.90(1.24–2.93)	0.003*	1.80(1.31–2.49)	< 0.001**
**Know where CCS is done**						
No	44(36.1)	21(12.0)	1.00			
Yes	78(63.9)	154(88.0)	2.05(1.30–3.24)	0.002*	1.99(1.42–2.81)	< 0.001**
**Partner support in HIV treatment**						
No	43(35.2)	53(30.3)	1.00			
Yes	77(63.2)	118(67.4)	1.10(0.79–1.52)	0.579		
No partner	2(1.6)	4(2.3)	1.21(0.44–3.34)	0.716		
**Embarrassed by the procedure**						
No	87(71.3)	130(74.3)	1.00			
Yes	35(28.7)	45(25.7)	0.94(0.67–1.32)	0.716		
**Fear outcomes of screening**						
No	63(51.6)	131(74.9)	1.00			
Yes	59(48.4)	44(25.1)	0.63(0.45–0.89)	0.009*	0.67(0.54–0.84)	< 0.001**
**Think screening is beneficial**						
No	21(17.2)	23(13.1)	1.00			
Yes	86(70.5)	147(84.0)	1.21(0.78–1.87)	0.402	0.85(0.65–1.12)	0.252
I don’t know	15(12.3)	5(2.9)	0.48(0.18–1.26)	0.135*	0.39(0.20–0.75)	0.005**
**Received HPV vaccination**						
No	76(62.3)	101(57.7)	1.00			
Yes	46(37.7)	74(42.3)	1.08(0.80–1.46)	0.612		
**Have friends/relatives who screened**						
No	61(50.0)	58(33.1)	1.00			
Yes	61(50.0)	117(66.9)	1.35(0.98–1.85)	0.063*	1.31(1.09–1.59)	0.005**
**Distance to the facility**						
Near	62(50.8)	88(50.3)	1.00			
Far	31(25.4)	50(28.6)	1.05(0.74–1.49)	0.774		
Very far	29(23.8)	37(21.1)	0.96(0.65–1.40)	0.817		
**Health education on CCS**						
No	51(41.8)	51 (29.1)	1.00			
Yes	71(58.2)	124(70.9)	1.27(0.92–1.76)	0.148*		
**CCS appointments at the facility**						
No	40(32.8)	41(23.4)	1.00			
Yes	82(67.2)	134(76.6)	1.23(0.86–1.74)	0.254		
**Conduct CCS outreaches**						
No	43(35.2)	39(22.3)	1.00			
Yes	75(61.5)	130(74.3)	1.33(0.93–1.91)	0.115*		
I don’t know	4(3.3)	6(3.4)	1.26(0.53–2.98)	0.596		
**Provider recommendation**						
No	26(21.3)	21(12.0)	1.00			
Yes	94(77.1)	152(86.9)	1.38(0.88–2.18)	0.164*		
I don’t know	2(1.6)	2(1.1)	1.12(0.26–4.77)	0.879		
**Convenient hours for CCS**						
No	47(38.5)	54(30.9)	1.00			
Yes	56(45.9)	110(62.9)	1.24(0.89–1.72)	0.196*		
I don’t know	19(15.6)	11(6.2)	0.69(0.36–1.31)	0.254		
**Availability of CCS services**						
No	15(12.3)	15(8.6)	1.00			
Yes	80(65.6)	144(82.3)	1.29(0.76–2.19)	0.133*		
I don’t know	27(22.1)	16(9.1)	0.74(0.75–2.18)	0.278		

Factors associated with the utilization of cervical cancer screening services among HIV-positive women aged 18–49 years. CCS- Cervical Cancer Screening, CPR- crude prevalence ratio at 95% confidence interval, APR- Adjusted prevalence ratio, CI- Confidence interval, *significant at *p* < 0.2. **significant at *p* < 0.05

## Discussion

Given that HIV-positive women bear a higher risk of developing cervical cancer, it was therefore crucial to investigate their level of utilization of cervical cancer screening services and associated factors at LRRH, Lira City, northern Uganda. Accordingly, our results indicate that only 58.9% of the HIV-positive women had screened for cervical cancer in the past 12 months and the predictors were ever heard of CCS, knowledge of where CCS is done, fear of CCS outcomes, not knowing whether CCS is beneficial or not and having friends/relatives who screened for cervical cancer. Our findings will help in the design and implementation of programs tailored to increasing cervical cancer awareness, and uptake of CCS and thereby reduce the disease burden.

In this study, 175(58.9%) participants had ever screened for cervical cancer in the past 12 months. The level of cervical cancer screening in this study could be explained by the fact that most women recruited were in HIV care and could have had more chances to get screened according to the WHO recommendations for screening and treatment of cervical cancer. Whereas, this level of uptake was higher than 43.75% and 30.3% in Uganda [23, 24], 46.3% in Kenya [26], and 40.1% in Addis Ababa [27]. The higher cervical cancer screening awareness among the participants in our study could have contributed to the higher level of utilization. However, the reported level is lower than the 80% target set by the Ministry of Health [28]. This is a depiction of the existing gaps in the implementation of the national strategic plan for cervical cancer prevention at the regional referral level where programs like health education/social mobilization, HPV vaccination, and CCS using Visual Inspection with Acetic Acid (VIA) or cytology have been under-utilized [28].

However, the prevalence in our study conforms with that in Canada (58%) [29], but is lower than that got from England (85.7%) [30]. The possible reason for the similarity could be due to increased access to CCS in both countries and the availability of specialist care at regional hospitals in Uganda [28]. The variation could be due to differences in the socio-demographic characteristics, socioeconomic status, and access to health among the respondents as well as the presence of more robust CCS programs in England. Furthermore, this discrepancy could be attributed to the uneven distribution of cervical cancer screening centers and the lack of integration of CCS services in HIV treatment centers. Thus, more HIV-positive women would benefit from CCS services if the screening guidelines for HIV-positive women are adhered to and integrated the services into routine HIV treatment services at all levels.

Our results indicate that participants never screened for cervical cancer because they didn’t have time to go for CCS, due to time inconvenience for the provision of CCS services at the facility, and because the procedure is painful. This depicts the effect of factors pertaining to the attitudes and perceptions of women towards CCS as they influence one’s intention to screen. Additionally, this explains the reasons why women present with late-stage disease that can hardly be treated even when the services are available. This is generally a cause of concern given the vulnerability to cervical cancer HIV-positive women bear. Besides, in the presence of a higher viral load, HPV persistence, and a positive margin status, there is an increased risk for recurrent cervical dysplasia resulting in invasive cervical cancers [31, 32]. Our finding conforms with previous studies which reported similar reasons for non-utilization of cervical cancer screening services [33, 34]. Continuous education on cervical cancer and screening is vital to enhance favorable attitudes toward CCS, increase risk perception, and address the fears held by women that would increase the demand and utilization of CCS.

Our study also found that women who were not sure whether CCS was beneficial were less likely to have screened compared to those who said it was not beneficial. This could be attributed to a lack of adequate knowledge of cervical cancer screening benefits and cervical cancer among those who were unsure [35–37]. On the other hand, those who thought screening was not beneficial could have screened due to other reasons such as peer influence, provider recommendation, and cervical cancer screening appointments [19, 38]. Being unsure of the benefits of CCS among women also corresponds to the negative attitudes towards screening reported in previous studies obtained from Ethiopia [36, 39] and Nigeria [40]. The finding in our study disagrees with those obtained from studies done in Latin America where women who knew the benefits of CCS had higher utilization [41, 42]. This variation could be due to differences in the awareness interventions as well as the socio-demographic characteristics between the participants. Thus, the provision of proper information regarding the benefits of cervical cancer screening would play a positive role in decision making which would increase utilization. During health education, health workers should emphasize the importance of screening early especially among women of reproductive age specifically on the poor obstetrical outcomes (preterm delivery, low birth weight, premature rupture of membranes) [43] following cancer treatment.

Our study also found that women who had ever heard about CCS were more likely to have screened compared to those who had never heard about CCS. This could be because HIV-positive women receive health education talks about cervical cancer and screening from their health facilities and are more likely to hear about CCS. Most participants reported that they received information regarding cervical cancer and screening from hospitals which could have influenced their cervical cancer screening practices, similar to previous studies [44, 45]. This finding conforms to that from previous studies in Tanzania [46], Uganda [47], and Ethiopia [39]. The provision of health education at every hospital visit and during routine care could improve the dissemination of information regarding cervical cancer and screening. Additionally, health workers can utilize media forums, outreach clinics in communities, and community drug distribution points to sensitize HIV-positive women about the importance of screening for cervical cancer as well as create cervical cancer awareness.

Additionally, the results of our study indicate that women who knew where CCS is done were more likely to have screened compared to their counterparts who did not know where CCS is done. The possible explanation could be that some of these women receive referrals as well as recommendations for CCS from their healthcare providers and peers. As a result, these women get to know where they can easily access affordable CCS services. Though it is not clear whether knowledge of a place for screening leads to CCS utilization, previous studies have shown positive associations between awareness of a place for screening and CCS utilization [19, 48]. Therefore, there should be a scaling up of CCS points and encourage eligible women to seek screening services from there. Additionally, as part of the bigger strategy, continuous awareness about the services, screening points as well and the time when the services are provided would leverage the uptake of CCS services among women.

In our study, women who feared the outcomes of CCS were less likely to have screened compared to those who did not fear the outcomes of cervical cancer screening. Fear of screening for CCS could result in HPV persistence which would eventually cause cervical dysplasia and cervical cancer [31]. Women could have feared screening outcomes due to misinformation regarding the harms and benefits of the CCS procedure as well as perceived bad outcomes such as serious side effects and positive results from the screening procedure. Part of the reason could be that in a low-resource setting where screening is not part of routine care [22], people are more likely to develop fear and anxiety about screening. This implies that in a setting where CCS services are routinely provided, the women are more likely to get used to screening which results in higher uptake. This can be possible if CCS is integrated into routine care up to the primary healthcare level. Furthermore, health education on CCS can include additional information on the benefits and risks associated with screening to enable clients to make informed choices. A similar finding was obtained from previous studies where women did not go for screening due to fear of test results [39, 47, 49].

Lastly, our findings in this study suggest that women who had friends and or relatives who had ever screened were more likely to have screened compared to those who did not have. This signifies the role played by peer and community networks in increasing CCS awareness and dispelling myths and misconceptions that exist in the community. It also implies that screening more women would have a positive influence on the screening behavior of other women who had never been screened previously. This claim is supported by previous studies that show an association between knowing someone who ever screened and utilization of CCS [19, 50]. This is because women who have been screened are more likely to have discussions on cervical cancer screening benefits and procedures with their peers. The finding underscores the importance of educating women in groups to facilitate understanding and discussions on cervical cancer and screening. Such strategies would enhance acceptability and willingness to screen for cervical cancer among women in different social settings.

### Study limitations and strengths

Cervical cancer screening utilization in this study was self-reported and was not validated by any backup records which could have introduced bias. Due to the small sample size used in this study, our findings may not be generalizable but rather transferable to similar settings. Furthermore, the study being cross-sectional made it difficult to assess causality between the dependent variable and independent factors. Additionally, self-reported responses could have introduced social desirability bias, especially on sensitive questions like the number of sexual partners and having been diagnosed with any sexually transmitted infections. Consecutive sampling used in this study, could have introduced selection bias since it denied participants an equal chance of participating in the study. Nevertheless, this study provides evidence on the level and factors associated with the utilization of cervical cancer screening services among HIV-positive women in the Lango subregion. The findings may contribute to the improvement of cervical cancer screening utilization among HIV-positive women.

### Conclusion and recommendations

The level of utilization of cervical cancer screening services among HIV-positive women was suboptimal. Implementation of structured interventions aimed at improving cervical cancer screening awareness among HIV-positive women is crucial. Additionally, to increase opportunities for screening and knowledge on cervical cancer prevention, screening and awareness programs can target HIV-positive women during their routine clinic visits.

Furthermore, health providers should ensure the provision of comprehensive health education on cervical cancer and screening including the harms and benefits of the procedures to influence the clients’ knowledge and positive attitudes. In addition, there is a need to utilize women’s social groups or organizations in communities to provide cervical cancer and CCS awareness. Future researchers should explore the perceptions and barriers to CCS utilization among women living with HIV.

### What is known about the topic

HIV-positive women are at a higher risk of developing cervical cancer because both HIV and HPV infections are transmitted sexually, and because HIV-induced immunosuppression increases the likelihood that HPV infection will persist in these women. In Uganda, there is poor integration of cervical cancer screening services in routine HIV care, with a lot of resource and funding gaps.

### What the study adds about the topic

The study highlights the level of utilization of cervical cancer screening services among women living with HIV in Lira Regional Referral Hospital.

## Data Availability

All the materials used in this study will be availed by the corresponding author upon request.

## Abbreviations

AIDS: Acquired immunodeficiency syndrome
ART: Antiretroviral therapy
CC: Cervical cancer
CCS: Cervical cancer screening
HIV: Human immunodeficiency virus
HPV: Human papillomavirus
LRRH: Lira regional referral hospital
MOH: Ministry of health
PAP: Smear papanicolaou smear
VIA: Visual inspection with acetic acid
